# Brain structural alterations and clinical features of cognitive frailty in Japanese community-dwelling older adults: the Arao study (JPSC-AD)

**DOI:** 10.1038/s41598-022-12195-4

**Published:** 2022-05-17

**Authors:** Kazuhiro Yoshiura, Ryuji Fukuhara, Tomohisa Ishikawa, Naoko Tsunoda, Asuka Koyama, Yusuke Miyagawa, Yosuke Hidaka, Mamoru Hashimoto, Manabu Ikeda, Minoru Takebayashi, Megumi Shimodozono

**Affiliations:** 1 Department of Neuropsychiatry, Kumamoto University Hospital, Kumamoto, Japan; 2 Department of Rehabilitation and Physical Medicine, Kagoshima University Graduate School of Medical and Dental Sciences, Kagoshima, Japan; 3 Department of Psychiatry, Kagoshima University Graduate School of Medical and Dental Sciences, Kagoshima, Japan; 4 Department of Geriatric Psychiatry, Mitsugumachi Clinic, Kumamoto, Japan; 5 Department of Neuropsychiatry, Faculty of Life Sciences, Kumamoto University, Kumamoto, Japan; 6 Department of Neuropsychiatry, Faculty of Medicine, Kindai University, Osaka-sayama, Japan; 7 Department of Psychiatry, Osaka University Graduate School of Medicine, Osaka, Japan

**Keywords:** Neuroscience, Health care, Medical research, Neurology, Risk factors, Signs and symptoms

## Abstract

Cognitive frailty (CF) is a clinical condition defined by the presence of both mild cognitive impairment (MCI) and physical frailty (PF). Elderly with CF are at greater risk of dementia than those with MCI or PF alone, but there are few known clinical or neuroimaging features to reliably distinguish CF from PF or MCI. We therefore conducted a population-based cross-sectional study of community elderly combining physical, cognitive, neuropsychiatric, and multisequence magnetic resonance imaging (MRI) evaluations. The MRI evaluation parameters included white matter (WM) lesion volumes, perivascular and deep subcortical WM lesion grades, lacunar infarct prevalence, microbleed number, and regional medial temporal lobe (MTL) volumes. Participants were divided into 4 groups according to the presence or absence of MCI and PF—(1) no MCI, PF (n = 27); (2) no PF, MCI (n = 119); (3) CF (MCI + PF) (n = 21), (4) normal controls (n = 716). Unique features of CF included shorter one-leg standing time; severe depressive symptoms; and MRI signs of significantly more WM lesions, lacunar infarcts, small-vessel disease lesions, microbleeds, and reduced MTL volumes. These unique deficits suggest that interventions for CF prevention and treatment should focus on motor skills, depressive symptoms, and vascular disease risk factor control.

## Introduction

The high-risk states of physical and cognitive decline termed physical frailty (PF) and mild cognitive impairment (MCI), respectively, have gained attention from researchers and clinicians interested in preventive medicine. They are age-related syndromes associated with higher risk of adverse events, such as dementia and death^1–3^. MCI has been identified as a prodromal stage of Alzheimer’s disease^4^. However, there is compelling evidence^5^ that such outcomes can be delayed by timely initiation of physical and cognitive rehabilitation regimens; therefore, it is imperative to identify reliable biomarkers for the “prodromal stage.” It is also vital to identify those individuals with MCI or PF at greatest risk for progression, and recent studies have suggested that both PF and MCI are important determinants of the transition to dementia^5^.

Physical and cognitive functions are closely related, and both physical and cognitive decline should be considered when assessing dementia risk^6,7^. PF is recognized as a factor that increases Alzheimer’s disease pathology, promotes more rapid cognitive decline, and influences dementia development^8,9^. The relatively new concept of “cognitive frailty” (CF) describes a heterogeneous clinical condition of aging that affects both physical and cognitive functions^10^. It has been suggested that CF is associated with a higher risk of dementia compared with that of MCI or PF alone^11^. Therefore, rehabilitation and other interventions during CF may be particularly valuable for reducing dementia incidence. To facilitate such interventions and delay the progression of dementia, it is necessary to identify the unique clinical features and risk factors distinguishing CF from PF and MCI^6^.

Four recent studies have investigated brain structural changes associated with CF using magnetic resonance imaging (MRI)^12–15^. These studies reported that compared to healthy adults without CF, those with CF had increased white matter hyperintensity lesions^12,13^ and greater volume reductions (atrophy) in the bilateral thalamus, left caudate, right pallidum, right accumbens^14^, and bilateral hippocampal subregions^15^. These lesion and atrophy patterns suggest that CF is a sign of progressive vascular pathology and neurodegenerative processes. However, the definition of CF varies among studies as there are no robust and widely accepted operational criteria. Previous studies have also measured the cognitive impairments of CF using a variety of screening tests that may emphasize dementia, depression, or clouded consciousness^16^. In most studies, CF is operationally defined as “reversible CF,” “potentially reversible CF,” or “potentially reversible CF International Academy of Nutrition and Aging (IANA)/International Association of Gerontology and Geriatrics (IAGG) model of CF^3^.” Reversible CF is a condition involving PF or pre-PF stage physical status (distinguished by the number of present frailty components among weakness, slowness, low activity, shrinking, and exhaustion) and preclinical cognitive decline, whereas potentially reversible CF involves pre-PF stage physical status (1 or 2 frailty components) and MCI stage cognitive status. “Potentially reversible CF IANA/IAGG model of CF” is defined by the IANA and IAGG international consensus group^10^ as PF stage physical status (≥ 3 frailty components) and MCI stage cognitive status (clinical dementia rating = 0.5)^17^. Among the three definitions, the third provides a stricter differentiation between CF and healthy older adults.

Previous brain imaging studies have defined CF according to original definitions^13–15^ or as “reversible cognitive deficits”^12^ rather than by a stricter operational definition that can reliably differentiate CF from PF, MCI, and normal aging groups. Therefore, we conducted a CF study using data from a population-based cross-sectional survey that defined CF according to the “potentially reversible CF IANA/IAGG model of cognitive frailty.” Also, rather than using an arbitrary screening value for MCI (clinical dementia rating = 0.5), MCI was determined by a physician trained to distinguish between MCI and other diseases like dementia and depression as described by Solffrizzi et al.^18^. We then compared the clinical features, such as motor performance, cognitive function, and depressive symptoms as well as brain structural alterations on MRI among individuals with CF, MCI alone, PF alone, or age-adjusted normal physical and cognitive functions. Based on previous CF study results, MRI structural evaluations conducted in our study targeted signs of small-vessel disease (SVD; e.g., white matter hyperintensity lesions), lacunar infarcts, microbleeds, and volume changes in regions implicated in dementia or Alzheimer’s disease, such as the medial temporal lobe (MTL).

## Results

### Demographic characteristics of the four clinical groups

**Figure 1:**
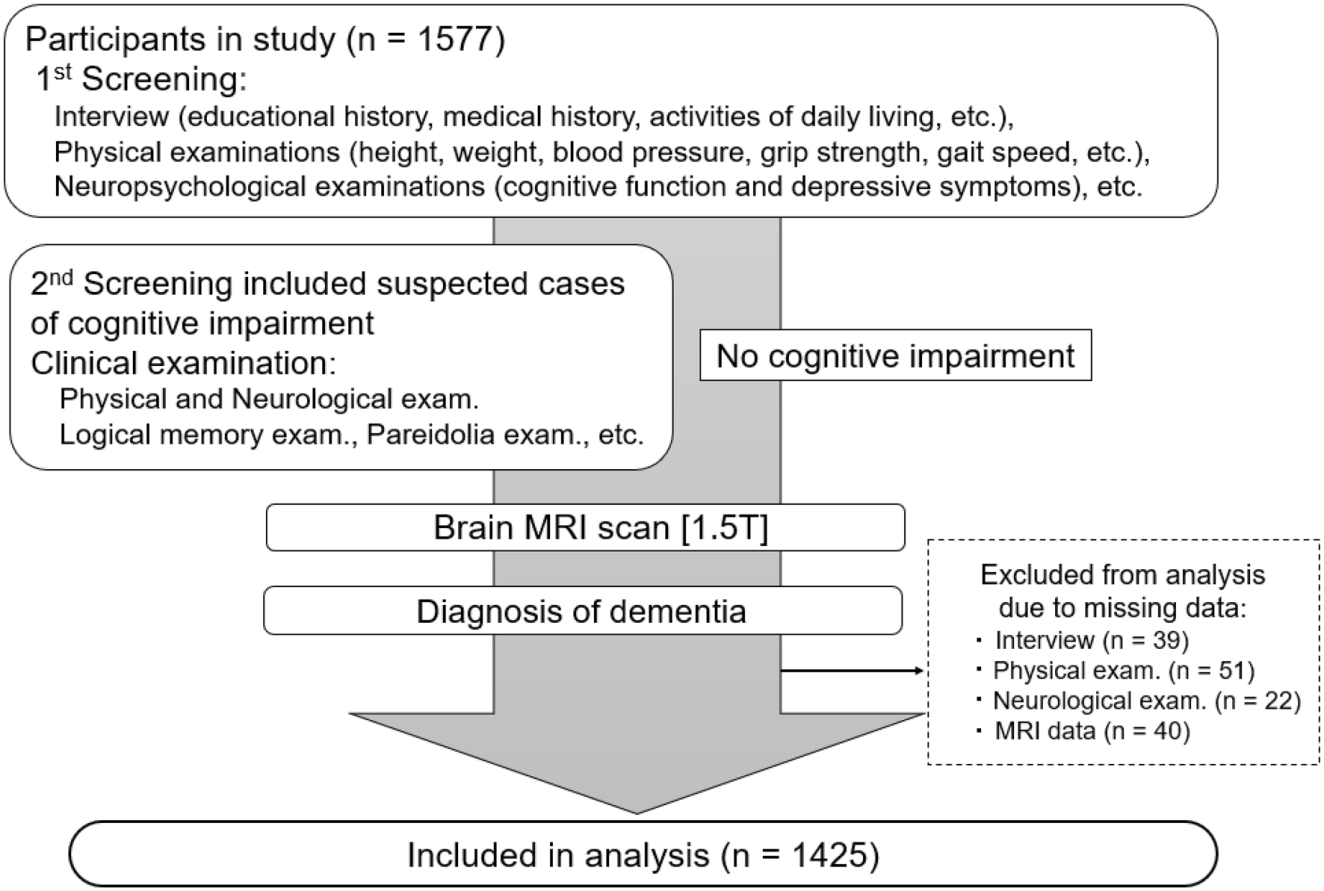
Flow diagram of study enrolment. The population-based cross-sectional data were obtained from one research site (Arao City, Kumamoto Prefecture) of the Japan Prospective Studies Collaboration for Aging and Dementia (JPSC-AD). A two-step screening process was then conducted to identify participants with physical frailty, mild cognitive impairment, concomitant physical frailty and mild cognitive impairment (“cognitive frailty”), and normal cognitive and physical functions. Participants diagnosed with dementia were excluded. *MRI* magnetic resonance imaging.

Of 1577 community-dwelling residents aged ≥ 65, 1425 individuals who agreed to participate in the survey and had no missing data were included in the analysis (Fig. 1). Table 1 shows the distribution of the participants stratified into nine categories according to the cognitive impairment criteria (i.e., no cognitive impairment, MCI, and dementia) and frailty (i.e., non-frail, pre-PF, and PF). Participants in this study were then divided into 4 groups according to the presence or absence of MCI and PF: (1) non-cognitively impaired PF (nci-PF), n = 27; (2) non-physically-frail MCI (npf-MCI), n = 119; (3) physical frailty and MCI (CF), n = 21; and (4) npf and nci (normal control: NC), n = 716. Table 2 presents the demographic characteristics of the four groups. The CF group had the highest mean age, whereas the CF and nci-PF groups exhibited higher Geriatric Depression Scale (GDS)-short version scores (indicative of more severe depressive symptoms)^19^ and lower motor function and Barthel Index (BI) scores than the npf-MCI and NC groups. There were no significant differences between the groups in terms of body mass index (BMI), prevalence of hypertension, and diabetes mellitus prevalence.

**Table 1:** Physical and cognitive status of individuals participating in the present survey.

	No cognitive impairment	Mild cognitive impairment	Dementia	Total
Non-frailty	**716 (50.2%)**	*119 (8.4%)*	13 (0.9%)	848 (59.5%)
Pre-frailty	404 (28.4%)	89 (6.2%)	25 (1.8%)	518 (36.3%)
Physical frailty	***27 (1.9%)***	21 (1.5%)	11 (0.8%)	59 (4.1%)
Total	1147 (80.5%)	229 (16.1%)	49 (3.4%)	1425 (100%)

Numbers and proportions (percentages) are presented corresponding to the physical status (i.e., non-frailty, pre-frailty, physical frailty) and cognitive status (i.e., no cognitive impairment, mild cognitive impairment, and dementia). Four subject groups selected for the present study are defined as follows: (a) cognitive frailty (CF), which was diagnosed in cases where both physical frailty and mild cognitive impairment criteria were met, n = 21, underlined in the table; (b) non-cognitive-impaired physical frailty (nci-PF), n = 27, bolditalics; (c) non-physically frail mild cognitive impairment (npf-MCI), n = 119, italics; (d) normal control (NC), i.e., non-physically-frail non-cognitive-impaired, n = 716, bold.

**Table 2:** Demographic characteristics of the participants in the four groups.

	CF (n = 21)	nci-PF (n = 27)	npf-MCI (n = 119)	NC (n = 716)	p-value
Age	83.1 ± 5.2	76.1 ± 8.1	76.2 ± 5.6	72.5 ± 5.4	< 0.001
Female	13 (61.9%)	21 (77.8%)	50 (42.0%)	424 (59.2%)	< 0.001
Education ≤ 9 years	10 (47.6%)	14 (51.9%)	45 (37.8%)	157 (21.9%)	< 0.001
Body mass index	22.7 ± 3.5	23.8 ± 4.3	23.5 ± 3.0	23.3 ± 3.0	0.509
One-leg standing time (s) ^a^	8.0 ± 7.0	25.2 ± 21.5	37.2 ± 21.4	46.2 ± 19.0	< 0.001
Timed up and go (s) ^b^	12.8 ± 3.0	12.3 ± 5.5	8.7 ± 1.7	8.1 ± 1.3	< 0.001
Gait speeds (m/s)	1.1 ± 0.3	1.2 ± 0.4	1.7 ± 0.3	1.8 ± 0.2	< 0.001
Grip strength (kg)	19.5 ± 6.3	19.6 ± 10.1	30.7 ± 8.5	29.6 ± 8.2	< 0.001
GDS score	4.7 ± 3.2	4.5 ± 3.1	2.6 ± 2.5	1.8 ± 1.9	< 0.001
GDS ≥ 6, GDS positive	8 (38.1%)	8 (29.6%)	18 (15.1%)	33 (4.6%)	< 0.001
MMSE score	23.4 ± 2.7	27.2 ± 2.4	24.6 ± 2.5	28.1 ± 2.0	< 0.001
Barthel Index	97.4 ± 5.8	97.2 ± 6.4	99.7 ± 2.1	99.8 ± 1.4	< 0.001
**Comorbidities and medications**					
History of stroke	3 (14.3%)	3 (11.1%)	8 (6.7%)	29 (4.1%)	0.042
History of cancer	3 (14.3%)	6 (22.2%)	11 (9.2%)	79 (11.0%)	0.264
History of respiratory disease	1 (4.8%)	4 (14.8%)	13 (10.9%)	56 (7.8%)	0.375
History of head injury	3 (14.3%)	0 (0.0%)	1 (0.8%)	16 (2.2%)	0.001
Hypertension^c^	19 (90.5%)	22 (81.5%)	85 (71.4%)	501 (70.0%)	0.128
Diabetes mellitus^d^	5 (23.8%)	8 (29.6%)	17 (14.3%)	109 (15.2%)	0.152
Depression^e^	0 (0.0%)	2 (7.4%)	0 (0.0%)	0 (0.0%)	< 0.001
Hypnotic and anti-anxiety drugs	3 (14.3%)	6 (22.2%)	17 (14.3%)	68 (9.5%)	0.080
**Frailty components**					
Weakness	17 (81.0%)	19 (70.4%)			n/a
Slowness	17 (81.0%)	19 (70.4%)			n/a
Low activity	16 (76.2%)	16 (59.3%)			n/a
Shrinking	14 (66.7%)	14 (51.9%)			n/a
Exhaustion	15 (71.4%)	15 (55.6%)			n/a

Data are presented as the mean ± standard deviation, or number and proportion (percentage). Categorical variables were compared among the four groups using the chi-square test, whereas continuous demographic variables were compared using one-way analysis of variance.

*CF* cognitive frailty, *nci-PF* non-cognitive-impaired physical frailty, *npf-MCI* non-physically-frail mild cognitive impairment, *NC* normal control (non-physically-frail non-cognitive-impaired individual group), *GDS* Geriatric Depression Scale (short version), *MMSE* Mini-Mental State Examination.

^a^The test was performed while the participants’ kept their eyes open. Left and right legs were assessed in the standing position, and the better (longer) record was used (maximum being 60 s).

^b^The test was performed at maximum walking speed.

^c^Hypertension was defined as blood pressure ≥ 140/90 mmHg or the use of antihypertensive agents.

^d^Diabetes was defined as fasting blood glucose ≥ 126 mg/dL, casual blood glucose ≥ 200 mg/dL, hemoglobin A1c ≧ 6.5%, or the use of glucose-lowering agents.

^e^Depression was diagnosed by a psychiatrist based on medical information and neuropsychiatric interviews according to the Diagnostic and Statistical Manual of Mental Disorders-fourth edition (DSM-IV).

### Unique clinical features of CF

CF group participants demonstrated significantly shorter one-leg standing times (OLSTs) (beta = − 18.2, SE = 4.3, p < 0.001) and lower Mini-Mental State Examination (MMSE)^20^ scores (beta = − 3.5, SE = 0.6, p < 0.001) than nci-PF group participants (Table 3). The CF group also demonstrated significantly shorter OLSTs (beta = − 26.7, SE = 2.6, p < 0.001) and weaker grip strength (beta = − 8.2, SE = 1.1, p < 0.001) than the npf-MCI group. In addition, CF group participants scored significantly higher (slower) on the Timed Up and Go (TUG) test (beta = 2.8, SE = 0.8, p < 0.001) and GDS-short version (beta = 3.3, SE = 1.1, p = 0.0030) than npf-MCI group participants. Compared to the participants in the NC group, those in the CF group had significantly shorter OLSTs (beta = − 40.4, SE = 1.2, p < 0.001) and lower gait speeds (beta = − 0.6, SE = 0.1, p < 0.001), exhibited weaker grip strength (beta = − 12.2, SE = 1.6, p < 0.001), had lower MMSE scores (beta = − 4.7, SE = 0.9, p < 0.001), longer (slower) TUG times (beta = 3.4, SE = 0.6, p < 0.001), and higher GDS scores (beta = 2.4, SE = 1.0, p = 0.015).

**Table 3:** Clinical features of each clinical group and pair-wise comparisons with the cognitive frailty group.

	CF (n = 21)	nci-PF (n = 27)	npf-MCI (n = 119)	NC (n = 716)	CF vs. nci-PF	CF vs. npf-MCI	CF vs. NC
One-leg standing time (s)	8.0 ± 7.0	25.2 ± 21.5	37.2 ± 21.4	46.2 ± 19.0	− 18.3*** (− 26.7, − 9.9)	− 28.7*** (− 33.8, − 23.6)	− 40.4*** (− 42.8, − 38.0)
Timed up and go (s)	12.8 ± 3.0	12.3 ± 5.5	8.7 ± 1.7	8.1 ± 1.3	− 1.5 (− 4.1, 1.2)	2.8*** (1.3, 4.3)	3.9*** (2.8, 5.1)
Gait speeds (m/s)	1.1 ± 0.3	1.2 ± 0.4	1.7 ± 0.3	1.8 ± 0.2	0.1 (− 0.2, 0.3)	0.0 (− 0.7, 0.6)	− 0.5*** (− 0.8, − 0.3)
Grip strength (kg)	19.5 ± 6.3	19.6 ± 10.1	30.7 ± 8.5	29.6 ± 8.2	− 0.2 (− 3.9, 3.5)	− 8.2** (− 11.2, − 5.2)	− 12.2*** (− 15.2, − 9.2)
MMSE score	23.4 ± 2.7	27.2 ± 2.4	24.6 ± 2.5	28.1 ± 2.0	− 3.5*** (− 4.8, − 2.3)	1.2 (− 0.9, 3.3)	− 4.7*** (− 6.5, − 3.0)
GDS score	4.7 ± 3.2	4.5 ± 3.1	2.6 ± 2.5	1.8 ± 1.9	0.1 (− 1.6, 1.4)	3.3** (1.1, 5.5)	2.4* (0.5, 4.3)

Data are presented as the mean ± standard deviation. For group comparisons, regression beta coefficients are presented with a 95% confidence interval.

*CF* cognitive frailty, *nci-PF* non-cognitive-impaired physical frailty, *npf-MCI* non-physically-frail mild cognitive impairment, *NC* normal control, *MMSE* Mini-Mental State Examination, *GDS* Geriatric Depression Scale (short version).

*p < 0.05, **p < 0.005, ***p < 0.001.

### Clinical features of nci-PF and npf-MCI

To confirm the clinical features of nci-PF and npf-MCI, supplementary comparisons were made between nci-PF, npf-MCI, and NC (Supplementary Table S1). Compared to the npf-MCI group, the nci-PF group demonstrated significantly lower gait speeds (beta = − 0.4, SE = 0.1, p < 0.001) as well as significantly higher TUG (beta = 3.1, SE = 0.7, p < 0.001), MMSE (beta = 2.5, SE = 0.7, p < 0.001), and GDS scores (beta = 1.6, SE = 0.5, p = 0.0020). Compared to NCs as well, nci-PF group participants had significantly lower gait speeds (beta = − 0.3, SE = 0.1, p = 0.001), longer TUG times (beta = 2.3, SE = 0.5, p < 0.001), and higher GDS scores (beta = 2.5, SE = 0.6, p < 0.001), and there was no difference in MMSE scores. Compared to NC group participants, the npf-MCI group participants had significantly lower MMSE (beta = − 3.2, SE = 0.3, p < 0.001) and significantly higher GDS (beta = 0.6, SE = 0.3, p = 0.019) scores.

### SVD pathology in CF

Table 4 summarizes the SVD features for each of the four groups revealed by MRI. Compared to nci-PF group participants, CF group participants exhibited higher white matter hypointensity^21^ volumes (beta = 9.37, SE = 3.84, p = 0.015), higher periventricular hyperintensity (PVH) grades (beta = 0.89, SE = 0.41, p = 0.029), and higher deep and subcortical white matter hyperintensity (DSWMH) grades (beta = 1.04, SE = 0.40, p = 0.010).Further, the proportion of subjects with ≥ 8 microbleeds was significantly greater in the CF group than the nci-PF group (beta = 21.14, SE = 0.59, p < 0.001). In addition, CF group participants had significantly higher grades of PVH (beta = 1.14, SE = 0.40, p = 0.0043) and DSWMH (beta = 1.18, SE = 0.40, p = 0.0030) and greater prevalence of lacunar infarcts (beta = 2.38, SE = 0.76, p = 0.0018) and ≥ 8 microbleeds (beta = 2.63, SE = 1.10, p = 0.016) than npf-MCI group participants. Compared with NCs, participants with CF also demonstrated significantly higher white matter hypointensity volumes (beta = 8.71, SE = 4.23, p = 0.040), PVH grades (beta = 1.13, SE = 0.56, p = 0.043), and DSWMH grades (beta = 1.21, SE = 0.54, p = 0.025) and higher prevalence of lacunar infarct (beta = 1.35, SE = 0.68, p = 0.047) and ≥ 8 microbleeds (beta = 2.46, SE = 0.78, p = 0.002). Refer to Supplementary Table S2 for the CF cerebrovascular disease profile.

**Table 4:** Small-vessel disease MRI features of each clinical group and pair-wise comparisons with the cognitive frailty group.

	CF (n = 21)	nci-PF (n = 27)	npf-MCI (n = 119)	NC (n = 716)	CF vs. nci-PF	CF vs. npf-MCI	CF vs. NC
White matter hypointensity (ml)	15.7 ± 15.9	6.3 ± 7.0	6.1 ± 5.4	4.1 ± 4.3	9.37* (1.84, 16.90)	11.07 (− 4.36, 26.51)	8.71* (0.41, 17.00)
PVH (grade 0–4)	2.3 ± 1.4	1.4 ± 1.1	1.3 ± 1.2	1.0 ± 1.1	0.89* (0.09, 1.69)	1.14*** (0.36, 1.92)	1.13* (0.04, 2.22)
DSWMH (grade 0–4)	2.4 ± 1.4	1.3 ± 1.1	1.3 ± 1.2	1.0 ± 1.1	1.04** (0.25, 1.83)	1.18*** (0.40, 1.96)	1.21* (0.15, 2.27)
Lacunar infarcts	11 (52.4%)	7 (25.9%)	31 (26.1%)	133 (18.6%)	0.55 (− 1.13, 2.13)	2.38*** (0.89, 3.87)	1.35* (0.02, 2.67,)
Microbleeds ≧ 1	7 (33.3%)	2 (7.4%)	17 (14.3%)	102 (14.2%)	0.66 (− 1.48, 2.80)	0.85 (− 0.96, 2.66)	1.06 (− 0.34, 2.46)
Microbleeds ≧ 8	4 (19.0%)	0 (0.0%)	3 (2.5%)	11 (1.5%)	21.14**** (19.99, 22.29)	2.63* (0.48, 4.79)	2.46*** (0.92, 3.99)

Data are presented as the mean ± standard deviation, or number and proportion (percentage). For group comparisons, regression beta coefficients are presented with a 95% confidence interval.

*CF* cognitive frailty, *nci-PF* non-cognitive-impaired physical frailty, *npf-MCI* non-physically-frail mild cognitive impairment, *NC* normal control, *PVH* periventricular hyperintensity, *DSWMH* deep and subcortical white matter hyperintensity.

*p < 0.05, **p < 0.01, ***p < 0.005, ****p < 0.001.

### Reduced MTL volume in CF

Table 5 shows the volumes of the four MTL structures for each of the four groups. Amygdalar volumes tended to be lower in the CF group than in the nci-PF group (beta = − 0.08, SE = 0.05, p = 0.084); no other measured MTL structure differed significantly in terms of volume between the CF and nci-PF groups. Similarly, no significant differences in MTL structure volumes were observed between the CF and npf-MCI groups. In contrast, compared to NCs, CF group participants exhibited significantly lower hippocampal (beta = − 0.68, SE = 0.16, p < 0.001), amygdalar (beta = − 0.17, SE = 0.04, p < 0.001), parahippocampal gyrus (beta = − 0.22, SE = 0.07, p < 0.001), and entorhinal cortex volumes (beta = − 0.20, SE = 0.08, p = 0.018).

**Table 5:** Medial temporal lobe volumes of each clinical group and pair-wise comparisons with the cognitive frailty group.

	CF (n = 21)	nci-PF (n = 27)	npf-MCI (n = 119)	NC (n = 716)	CF vs. nci-PF	CF vs. npf-MCI	CF vs. NC
Hippocampus (ml)	3.0 ± 0.5	3.4 ± 0.4	3.4 ± 0.5	3.7 ± 0.4	− 0.27 (− 0.59, 0.06)	− 0.02 (− 0.30, 0.26)	− 0.68** (− 0.99, − 0.38)
Amygdala (ml)	1.0 ± 0.2	1.2 ± 0.1	1.2 ± 0.2	1.2 ± 0.2	− 0.08 (− 0.18, 0.01)	0.02 (− 0.14, 0.19)	− 0.17** (− 0.25, − 0.09)
Parahippocampal gyrus (ml)	1.5 ± 0.2	1.6 ± 0.3	1.6 ± 0.3	1.7 ± 0.3	0.02 (− 0.12, 0.17)	0.07 (− 0.24, 0.37)	− 0.22** (− 0.35, − 0.09)
Entorhinal cortex (ml)	1.6 ± 0.3	1.7 ± 0.3	1.7 ± 0.4	1.8 ± 0.3	− 0.08 (− 0.25, 0.09)	0.19 (− 0.08, 0.45)	− 0.20* (− 0.37, − 0.03)

The average volumes of the left and right regions were calculated. Data are presented as the mean ± standard deviation. For group comparisons, regression beta coefficients are presented with a 95% confidence interval.

*CF* cognitive frailty, *nci-PF* non-cognitive-impaired physical frailty, *npf-MCI* non-physically-frail mild cognitive impairment, *NC* normal control.

*p < 0.05, **p < 0.001.

### MRI features of nci-PF and npf-MCI

Supplementary comparisons were made between nci-PF, npf-MCI, and NC groups (Supplementary Table S3) to confirm the MRI features of nci-PF and npf-MCI. Compared to the npf-MCI group, the nci-PF group demonstrated a significantly lower prevalence of ≥ 8 microbleeds (beta = − 18.82, SE = 0.63, p < 0.001) and a trend for higher amygdalar volumes (beta = 0.05, SE = 0.03, p = 0.092). Compared to NCs, the nci-PF group exhibited a lower prevalence of ≥ 1 microbleeds (beta = − 2.90, SE = 0.91, p = 0.0014) and ≥ 8 microbleeds (beta = − 19.29, SE = 0.45, p < 0.001) as well as lower entorhinal cortex volumes (beta = − 0.12, SE = 0.06, p = 0.042). Compared to the NC group, the npf-MCI group exhibited significantly lower hippocampal (beta = − 0.19, SE = 0.07, p = 0.0063), amygdalar (beta = − 0.05, SE = 0.02, p = 0.023), parahippocampal gyrus (beta = − 0.06, SE = 0.03, p = 0.049), and entorhinal cortex volumes (beta = − 0.10, SE = 0.04, p = 0.010) as well as a trend for higher white matter hypointensity volume (beta = 0.82, SE = 0.49, p = 0.099).

## Discussion

To identify unique clinical and neuroimaging features of CF (e.g., white matter hypointensity, PVH, DSWMH, lacunar infarcts, microbleeds, and MTL volume), we selected individuals meeting strict diagnostic criteria for PF, MCI, or both (the operational definition of CF according to the IANA/IAGG working group) from a population-based survey (those meeting no such criteria were included as healthy controls); conducted a comprehensive battery of physical, cognitive, and neuropsychiatric tests as well as multisequence MRI; compared the results of the four groups; and also selected data from the population-based survey.

Performance on the OLST was significantly poorer in the CF group compared to the other three groups, whereas depressive symptoms as measured by GDS scores were more severe in the CF group compared to the npf-MCI and NC groups. Further, brain MRI evaluation revealed substantially more advanced signs of SVD in the CF group than in the other groups. Specifically, the CF group had more white matter hypointensity, DSWMH, and microbleeds than the nci-PF group; more PVH, DSWMH, lacunar infarcts, and microbleeds than the npf-MCI group; and more white matter hypointensity, PVH, DSWMH, lacunar infarcts, and microbleeds than the NC group. Consistent with more advanced SVD and concomitant brain atrophy underlying cognitive dysfunction, CF group also exhibited reduced hippocampus, amygdala, parahippocampal gyrus, and entorhinal cortex volumes compared to the NC group, and numerically lower volumes compared to npf-MCI and nci-PF groups. Thus, the CF group demonstrated the same (high-level) decline in motor skills and an increase in depressive symptoms compared to the nci-PF group and the same level of MTL atrophy and cognitive decline as the npf-MCI group. The CF group was unique, showing signs of severe SVD. The nci-PF and npf-MCI groups did not have more white matter hypointensity, PVH, DSWMH, lacunar infarcts, and microbleeds (Supplementary Table S3) compared with the NC group.

A previous longitudinal study reported that reversible CF, defined by PF status and subjective cognitive decline, was specifically associated with vascular dementia incidence^22^. The conspicuous absence of SVD in the nci-PF and npf-MCI groups seemingly contradicts reports of significant associations between SVD and PF and MCI in previous studies^23,24^. However, when interpreting previous study results regarding the brain structural alterations or clinical features, we may have to reconsider the differences in the definitions of PF and MCI used in individual studies, which may have led to the possible inclusion of individuals with cognitive impairment in the PF group and/or inclusion of individuals with pre-PF or PF in the MCI group, resulting in the inadvertent inclusion of individuals with CF in other groups. In the present study, the CF group showed more severe signs of SVD than the other three groups; therefore, we believe that the inclusion of participants with CF in the “MCI” and “PF” groups in previous studies may have led to spurious associations of “MCI” and “PF” with SVD.

In this study, we detected significant MTL region volume loss in the CF group, comparable to that observed in the npf-MCI group. Cognitive decline in the CF and npf-MCI group participants as measured by the MMSE may reflect Alzheimer’s disease pathology, which is known to involve MTL atrophy, similar to the MCI mechanism. In contrast, OLST was the only physical performance metric that was significantly reduced in CF compared to the other three groups. A previous study reported a significant association between short OLSTs and MCI, and the combination of short OLST with decreased grip strength (a weakness frailty component) further strengthened the association^25^. These findings are consistent with our results showing that the CF group (i.e., PF and MCI) had short OLST. Moreover, OLST performance was found to be associated with asymptomatic SVD in previous brain MRI studies^26^. Short OLSTs among CF group participants in this study may indicate the presence of significantly more advanced SVD in them compared to participants with MCI or PF alone. In our investigation, GDS scores were also significantly higher in the CF group than in the npf-MCI and NC groups, consistent with a previous report in which participants with both PF and cognitive impairments, including dementia, had significantly higher scores for another depression screening instrument, the Patient Health Questionnaire-2, compared to those in the non-impaired group^27^. The present findings that SVD and depressive symptoms are characteristic of CF may also be supported by biological studies^28,29^ showing that neuroinflammatory markers associated with SVD and depression (e.g., interleukin-6) are also biomarkers for CF.

In this study, we did not observe severe SVD in the npf-MCI group, which is an important finding for dementia prevention. The development of MCI is significantly associated with MTL region atrophy, and it has been suggested that SVD, such as white matter hyperintensity lesions and lacunar infarctions, are associated with the transition from MCI to dementia^30^. Therefore, interventions targeting PF components in older adults to reduce SVD risk factors may prevent or delay the onset of dementia.

The present study had several limitations. First, two different models of MRI machines were used in this population-based survey; thus, it is possible that the MRI data differed among models. However, we employed measures to correct geometric distortions^31^, and there were no significant differences in the proportion of each group analyzed by one system or the other. Second, this study had a small sample size (especially the CF group), which may have reduced the statistical power of the analyses. Third, we used the inverse propensity weighting propensity score (IPW-PS) method for statistics, which while advantageous for adjusting confounding factors in a limited number of samples, limits multiple comparisons^32^. Nonetheless, we were able to adjust for the degree of impairment in the analyses of CF vs. nci-PF and CF vs. npf-MCI by including the number of frailty component defects and MMSE scores in the propensity score. In recent reports^33,34^, multiple propensity score analysis has been used to compare multiple groups, but in order to understand the characteristics of CF, we chose to analyze each of the two groups adjusted for the degree of impairments. Due to the difference of independent variables used in IPW-PS analysis, we did not correct for multiple comparisons. More rigorous corrections with a larger sample size will be required to corroborate our results. Fourth, we excluded the data of individuals that did not have all the data components required for analysis, which may have introduced selection bias due to elimination of individuals with severe disabilities or depression (e.g., individuals unable to walk or tolerate MRI conditions). Finally, our analyses included subjects with histories of stroke or head injury (Table 2). However, MRI evaluations of 21 participants with CF showed no direct damage, at least of the pyramidal tract, caused by cerebral hemorrhage, infarction, or injury. Therefore, we included histories of stroke and head injury as independent variables to calculate the propensity scores.

To our knowledge, this is the first imaging study that comprehensively evaluates brain lesions predictive of SVD, such as white matter hypointensity, PVH, DSWMH, lacunar infarction prevalence, microbleed number, and MTL volume in elderly with CF. Although previous studies have indicated that white matter hyperintensity lesions are associated with CF, this is the first report of a population-based study. In addition, we analyzed SVD in more detail than in previous studies^13–15^, including evaluations of lacunar infarcts and microbleeds. We also examined MTL region volumes because volume reductions have been linked to early pathological changes in Alzheimer’s disease, the most frequent dementia to develop from amnestic MCI. Moreover, multisequence brain MRI enables concurrent assessments of brain structural alterations and clinical symptoms. One of the limitations of postmortem-based brain research^8,9^ is that the effects of terminal decline or causes of death other than dementia are not considered. Another strength of this study was the use of more rigorous criteria to define CF characteristics than those used in previous reports. Specifically, the CF group excluded those in the pre-PF stage, and MCI was based on diagnosis made by a clinical psychiatrist instead of a scale (i.e., clinical dementia rating = 0.5)^18^. Further, all participants were examined by senior neuropsychiatrists, and participants with probable dementia were strictly excluded.

In conclusion, the CF group exhibited motor performance declines similar to those observed in PF and cognitive declines and MTL region atrophies similar to those observed in MCI. The unique aspects of CF were poorer OLST performance, severe depressive symptoms, and imaging signs of severe SVD (higher white matter hypointensity volume, PVH grade, DSWMH grade, lacunar infarct prevalence, and microbleed number). Therefore, older adults with CF are considered to be at very high risk for conversion to dementia due to increased Alzheimer’s and cerebrovascular pathologies than those with PF or MCI alone. Most participants with CF in this study were community-dwelling older adults who maintained independent activities of daily living (age: 83.1, BI: 97.1 points). Therefore, appropriate preventive intervention for CF may delay or prevent dementia onset in such populations. For maintaining the independence of individuals with CF in performing daily living activities, factors such as physical performance, depressive symptoms, and vascular disease risk should be considered. Further longitudinal studies to validate the effectiveness of such interventions are warranted.

## Methods

### Study design and participants

Data were obtained from a subset (one research site) of the Japan Prospective Studies Collaboration for Aging and Dementia (JPSC-AD), a population-based prospective cohort study to establish preventive strategies for dementia^31^. The JPSC-AD surveys community-dwelling residents aged ≥ 65 from eight research sites in Japan. The current study used baseline data of 1577 elderly individuals surveyed in Arao City, Kumamoto Prefecture, from 2016 to 2017. These data were acquired in compliance with relevant institutional and national guidelines and regulations. The current study was approved by the ethics committee of Kumamoto University (GENOME-333). Participants were informed of the purpose and methods of this study, and written informed consent was obtained. If participants were unable to provide consent for any reason, such as severe dementia, informed consent was obtained from a family member or other agent.

### Procedure

This study was conducted using procedures designed by researchers in dementia medicine from the JPSC-AD group to accurately diagnose and distinguish elderly individuals with cognitive impairments such as MCI and dementia. Figure 1 shows the participant selection flow chart. In the first screening phase, we interviewed all participants and performed physical and neuropsychological examinations. Participants meeting the following cognitive impairment criteria were then selected for further study: (1) MMSE score ≤ 26, (2) score ≤ 4 out of 6 on the MMSE delayed recall component (consisting of 3 questions, each scored 2 points if answered correctly without a hint, 1 point if answered correctly with a hint, and 0 points if answered incorrectly), (3) failed the intersecting pentagon copying component of the MMSE or made errors on the cube copying test, (4) suspected cases based on speaking manner and/or behavior (based on criteria from a previous study^31^). Next, we performed MRI scans on almost all selected individuals except those with a confirmed diagnosis of dementia (See “Dementia and MCI diagnoses”). We also excluded participants with any missing baseline data, resulting in the inclusion of 1,425 participants in the analysis.

### PF and pre-PF assessment

Pre-PF and PF were diagnosed using the Cardiovascular Health Study (CHS) criteria^35^, where pre-PF is defined by the presence of one or two of the following five components: weakness, slowness, low activity, shrinking, and exhaustion, whereas PF is defined by the presence of three or more of those components. In the present study, we used the Japanese version of the CHS criteria^36^, which uses a revised definition for weakness^37^ (grip strength < 28 kg for males and < 18 kg for females). Grip strength was measured using a TOEI LIGHT T2177 Hand Strengthener Grip D (TOEI LIGHT, Souka City, Saitama, Japan). The time required to walk 5 m was used as an index of slowness, with comfortable gait speed < 1.0 m/s defined as slow. Low activity was defined by “no” answers for two questions: (1) “Do you engage in moderate levels of physical exercise or sports aimed at health?” and (2) “Do you engage in low levels of physical exercise aimed at health?” Participants answering “yes” to the question “Have you (unintentionally) lost weight ≥ 2–3 kg in the past 6 months?” were classified as shrinking. Finally, exhaustion was assessed using the Japanese version of the Patient Health Questionnaire-9^38^ (PHQ-9). Exhaustion was defined as answering “More than half the days” or “Nearly every day” to the item “(Over the last 2 weeks) Feeling tired or having little energy.”

### Dementia and MCI diagnoses

Dementia or MCI were diagnosed in two steps according to the method specified by JPSC-AD^31^. Expert psychiatrists made diagnoses based on physical and neurological examinations, interviews with family members and attending physicians, medical records, and brain imaging. Additionally, psychiatrists or neurologists at a different research site diagnosed all cases of cognitive impairment after reviewing the clinical evaluation. If the diagnoses were inconsistent, an endpoint adjudication committee confirmed the diagnosis through discussion. Dementia was diagnosed according to the Diagnostic and Statistical Manual of Mental Disorders, 3rd Revised Edition (DSM-III-R), and MCI was diagnosed according to the criteria of Petersen et al.^2^.

### CF and NC diagnoses

CF was diagnosed in cases where both PF and MCI criteria were met. The definition of cognitive decline was based on MCI diagnosis. We referred to three reports regarding CF diagnosis. First, among the various diagnostic criteria for CF, the combination of PF and MCI carries a higher risk of dementia and early mortality^11^. Second, PF is associated with more severe cognitive impairment than pre-PF^39^. Third, MCI diagnosis can distinguish between pre-dementia and dementia more accurately than the “clinical dementia rating = 0.5” criterion^18^. Participants who did not meet all PF, pre-PF, dementia, and MCI criteria constituted the NC group.

### MRI data acquisition

Brain MRIs were conducted at Arao Municipal Hospital using a 1.5-Tesla Philips Ingenia CX Dual Scanner (Philips Healthcare, Best, Netherlands) and at Omuta Tenryo Hospital using a 1.5-Tesla GE Signa HDxt Ver.23 Scanner (GE Healthcare, Milwaukee, USA). The Philips MRI scanning protocol consisted of a three-dimensional (3D) T1-weighted sequence (repetition time = 8.6 ms, echo time = 4.0 ms, flip angle = 9°, matrix = 192 × 192, slice thickness = 1.2 mm), 3D T2-weighted sequence (repetition time = 5082.3 ms, echo time = 100.0 ms, flip angle = 90°, matrix = 356 × 248, slice thickness = 5.0 mm), 3D fluid-attenuated inversion recovery (FLAIR) sequence (repetition time = 11,000.0 ms, echo time = 120.0 ms, flip angle = 90°, matrix = 288 × 203, slice thickness = 5.0 mm), and susceptibility-weighted imaging (SWI) sequence (repetition time = 78.4 ms, echo time = 41.4 ms, flip angle = 20°, matrix = 288 × 272, slice thickness = 2.4 mm). The GE Signa MRI scanning protocol consisted of a 3D T1-weighted sequence (repetition time = 8.3 ms, echo time = 3.4 ms, flip angle = 8°, matrix = 192 × 192, slice thickness = 1.2 mm), 3D T2-weighted sequence (repetition time = 4517.0 ms, echo time = 92.6 ms, flip angle = 90°, matrix = 352 × 224, slice thickness = 5.0 mm), 3D FLAIR sequence (repetition time = 10,000.0 ms, echo time = 149.2 ms, flip angle = 90°, matrix = 288 × 193, slice thickness = 5.0 mm), and 3D T2-star weighted angiography sequence (repetition time = 75.2 ms, echo time = 57.9 ms, flip angle = 20°, matrix = 320 × 200, slice thickness = 3.0 mm).

### Brain MRI SVD evaluations

Images were analyzed by one neuroradiologist and one neuropsychiatrist in accordance with Japanese guidelines^40^. White matter hypointensity, PVH, DSWMH, lacunar infarcts and microbleeds were evaluated by visual inspection of T1-weighted, T2-weighted, FLAIR, and SWI images^41^. We defined two categories of microbleeds according to previous reports, one or more and eight or more^42,43^. The severities of PVH and DSWMH were graded on a 0–4 scale according to the report by Shinohara et al^44^.

### Image processing

Volume segmentation was performed on T1-weighted images using CentOS 6 Free-Surfer 5.3 (http://surfer.nmr.mgh.harvard.edu/) as previously reported^45–47^. The volumes of the hippocampus, amygdala, parahippocampal gyrus, and entorhinal cortex were defined according to the Desikan-Killany Atlas^48^ and subcortical structures according to Fischl et al.^49^. The volume of each region was calculated as the mean of the left and right sides of the brain.

### Physical performance

OLST was performed twice on each side with eyes open, and the longest time taken was accepted as the performance index for analysis (with a maximum limit of 60 s). The TUG test was also measured twice, and the shortest time was accepted as the performance index. Gait speed was calculated by measuring the time required to walk 5 m as fast as possible (m/s). Grip strength was measured twice on each side, and the highest score was included in the analysis. Physical performance tests were performed by trained physical and occupational therapists.

### Cognitive and depressive statuses

Cognitive and depressive statuses were evaluated by trained clinical psychologists and psychiatrists. Cognitive function was evaluated using the MMSE and depression using GDS. In the Japanese version of GDS, a score ≥ 6 indicated depressive symptoms^50^.

### Statistical analysis

Categorical demographic variables were compared among groups using the chi-square test and continuous demographic variables using one-way analysis of variance. Physical performance scores/times, brain structure volumes, grading scale scores, and lesion numbers were compared among groups (i.e., CF vs. nci-PF, CF vs. npf-MCI, CF vs. NC) using inverse probability weighting of propensity scores (IPW-PS). Propensity scores were calculated by binomial logistic regression with independent variables that differed for each analysis, considering the characteristics of two groups being compared as follows. (1) Independent variables for the CF vs. nci-PF comparison were age, sex, education, BI, BMI, history of stroke, history of head injury, depression, use of hypnotic and anti-anxiety drugs, and estimated total intracranial volume (the above is a total of “10 variables”), plus the total number of frailty components (0–5 defects) to adjust for the effects of frailty severity, for a total of 11 variables. (2) Independent variables for the CF vs. npf-MCI comparison were MMSE score (0–30 points) to adjust for the effects of cognitive impairment severity in addition to the same “10 variables” as mentioned above, for a total of 11 variables. (3) Independent variables for the CF vs. NC comparison were the “10 variables” common to CF vs. nci-PF, and CF vs. npf-MCI. We also calculated the average treatment effect (ATE) weight using propensity scores and compared groups weighted by ATE using a generalized linear model. A p < 0.05 was considered statistically significant for all tests. All analyses were conducted using IBM SPSS Statistics 25.0 Armonk, NY: IBM Corp. (https://www.ibm.com/analytics/spss-statistics-software).

## Supplementary Information

Supplementary Tables.

## Data Availability

All the processed data generated during this study are provided in the main article and Supplementary Information. The raw data is not openly available to protect the confidentiality of participants and to comply with the terms of participant consent. Requests related to the raw data should be addressed to the corresponding author.
