# Supplementary material for: Brain structural alterations and clinical features of cognitive frailty in Japanese community-dwelling older adults: the Arao study (JPSC-AD)
Source: Sci Rep. 2022 May 17;12:8202. doi: 10.1038/s41598-022-12195-4 (PMC9114363; doi:10.1038/s41598-022-12195-4)
Supplement: Supplementary file 1 — Supplementary Tables. [file 41598_2022_12195_MOESM1_ESM.pdf]

Brain structural alterations and clinical features of cognitive frailty in Japanese community-dwelling older adults: The Arao study (JPSC-AD)

Kazuhiro Yoshiura, Ryuji Fukuhara, Tomohisa Ishikawa, Naoko Tsunoda, Asuka Koyama, Yusuke Miyagawa, Yosuke Hidaka, Mamoru Hashimoto, Manabu Ikeda, Minoru Takebayashi, Megumi Shimodozono

Supplementary information

Table of contents

Supplementary Table S1 2
Supplementary Table S2 3
Supplementary Table S3 4

Supplementary Table S1. Clinical features and comparisons among non-cognitive-impaired physically frail, non-physically-frail mild cognitive impairment, and normal control groups

	nci-PF vs. npf-MCI	nci-PF vs. NC	npf-MCI vs. NC
One-leg standing time (s)	−7.7 (−20.1, 4.7)	−4.2 (−16.3, 8.0)	−3.7 (−8.7, 1.2)
Timed up and go (s)	3.1*** (1.7, 4.5)	2.3*** (1.3, 3.3)	0.3 (−0.1, 0.7)
Gait speeds (m/s)	−0.4*** (−0.5, −0.2)	−0.3** (−0.4, −0.1)	0.0 (−0.1, 0.0)
Grip strength (kg)	−6.3 (−14.0, 1.4)	−2.8 (−14.5, 8.9)	−0.4 (−2.4, 1.6)
MMSE score	2.5*** (1.1, 3.8)	0.2 (−0.9, 1.3)	−3.2*** (−3.8, −2.6)
GDS score	1.6** (0.6, 2.6)	2.5*** (1.3, 3.7)	0.6* (0.1, 1.1)

For group comparisons, regression beta coefficients are presented with a 95% confidence interval. *$p < 0.05$, **$p < 0.005$, *** $p < 0.001$. nci-PF, non-cognitive-impaired physical frailty; npf-MCI, non-physically-frail mild cognitive impairment; NC, normal control; MMSE, Mini-Mental State Examination; GDS, Geriatric Depression Scale.

Supplementary Table S2. Cerebral vascular disease profiles of participants with cognitive frailty

Case (No.)	Age (range)	Sex	Microbleeds [Lobar]		Microbleeds [Deep or infratentorial]		Lacunar infarcts		Old infarcts		PVH	DSWMH
1	75–79	M	+	frontal<br>temporal<br>occipital	+	amygdala<br>pallidus<br>thalamus<br>hippocampus<br>brain stem	+	periventricular<br>putamen<br>thalamus	-		+	+
2	75–79	M	-		-		-		-		+	+
3	75–79	M	+	frontal	+	putamen<br>thalamus<br>cerebellum	+	putamen<br>frontal<br>parietal	-		+	+
4	75–79	F	-		+	pallidus	+	periventricular<br>putamen	+	temporal	+	+
5	75–79	F	-		-		-		-		+	+
6	75–79	F	-		-		-		-		-	-
7	75–79	M	+	frontal<br>temporal<br>occipital	+	putamen<br>thalamus	+	periventricular<br>putamen	-		+	+
8	75–79	M	-		-		+	putamen<br>caudate	-		+	+
9	80–84	M	-		-		-		-		-	-
10	80–84	M	-		-		-		-		+	+
11	80–84	F	-		-		+	putamen	-		+	+
12	85–89	F	-		-		-		-		+	+
13	85–89	F	-		-		-		-		+	+
14	85–89	F	+	frontal<br>parietal<br>temporal<br>occipital	+	deep white matter<br>putamen<br>thalamus<br>brain stem<br>cerebellum	+	periventricular<br>putamen<br>thalamus<br>pallidus<br>caudate<br>brain stem	-		+	+
15	85–89	F	-		-		+	periventricular<br>putamen	+	frontal	+	+
16	85–89	F	+	temporal<br>occipital	+	brain stem<br>cerebellum	-		-		+	+
17	85–89	F	-		-		-		-		+	+
18	85–89	F	-		-		+	periventricular	-		+	+
19	85–89	F	-		-		-		-		+	+
20	90–94	F	+	frontal	-		+	periventricular	-		+	+
21	90–94	M	-		-		-		-		+	+

PVH, periventricular hyperintensity; DSWMH, deep and subcortical white matter hyperintensity.

Supplementary Table S3. Small-vessel disease MRI features, medial temporal lobe volumes, and comparisons among non-cognitive-impaired physically frail, non-physically-frail mild cognitive impairment, and normal control groups

		nci-PF vs. npf-MCI	nci-PF vs. NC	npf-MCI vs. NC
SVD pathology	White matter hypointensity (ml)	−0.26 (−2.98, 1.39)	−0.07 (−1.99, 1.86)	0.82 (−0.15, 1.78)
	PVH (grade 0–4)	−0.15 (−0.65, 0.25)	−0.17 (−0.70, 0.37)	0.13 (−0.12, 0.38)
	DSWMH (grade 0–4)	−0.15 (−0.69, 0.28)	−0.25 (−0.84, 0.35)	0.15 (−0.12, 0.42)
	Lacunar infarcts	−0.50 (−1.61, 0.56)	−0.61 (−2.08, 0.87)	0.05 (−0.48, 0.57)
	Microbleeds ≥ 1	−1.08 (−2.87, 0.92)	−2.90*** (−4.69, −1.12)	−0.44 (−1.07, 0.19)
	Microbleeds ≥ 8	−18.82*** (−20.06, −17.58)	−19.29*** (−20.18, −18.41)	0.40 (−1.02, 1.83)
MTL volume	Hippocampus (ml)	0.13 (−0.04, 0.08)	−0.07 (−0.20, 0.06)	−0.19** (−0.32, −0.05)
	Amygdala (ml)	0.05 (−0.01, 0.03)	0.01 (−0.06, 0.07)	−0.05* (−0.09, −0.01)
	Parahippocampal (ml)	−0.01 (−0.13, 0.06)	−0.05 (−0.20, 0.09)	−0.06* (−0.12, 0.00)
	Entorhinal (ml)	0.05 (−0.11, 0.08)	−0.12* (−0.24, 0.00)	−0.10* (−0.18, −0.02)

For group comparisons, regression beta coefficients are presented with a 95% confidence interval. *p < 0.05, **p < 0.01, ***p < 0.005. nci-PF, non-cognitive-impaired physical frailty; npf-MCI, non-physically-frail mild cognitive impairment; NC, normal control; SVD, small-vessel disease; MTL, medial temporal lobe; PVH, periventricular hyperintensity; DSWMH, deep and subcortical white matter hyperintensity; Parahippocampal, parahippocampal gyrus; Entorhinal, entorhinal cortex.
